# Bioelectrical impedance spectroscopy can assist to identify the parathyroid gland during thyroid surgery

**DOI:** 10.3389/fendo.2022.963520

**Published:** 2022-09-15

**Authors:** Bin Wang, Zaoyang Liu, Jian Wu, Ying Liu, Pin Wang, Hong Liu, Haobin Wang, Tielin Wang, Juan Wang, Yan Tang, Junyan Zhang

**Affiliations:** ^1^ Center of Breast and Thyroid Surgery, Department of General Surgery, Chengdu Third People’s Hospital, Chengdu, China; ^2^ Department of General Thoracic Surgery, Chengdu Third People’s Hospital, Chengdu, China; ^3^ Department of Ultrasound, Chengdu Third People’s Hospital, Chengdu, China; ^4^ Department of Pathology, Chengdu Third People’s Hospital, Chengdu, China; ^5^ Department of Computer Science, George Washington University, Washington, DC, United States

**Keywords:** bioelectrical impedance spectroscopy, thyroid, parathyroid gland, lymph node, adipose tissue

## Abstract

**Objective:**

This study aimed to explore the effectiveness of bioelectrical impedance spectroscopy in the identification of parathyroid glands during thyroid surgeries.

**Method:**

All patients who received thyroid surgeries at our department from January 2018 to February 2020 were recruited for this study. The bioelectrical impedance spectroscopy analyzer was applied to analyze on following tissues: thyroid tissues, lymph nodes, adipose tissues, and the tissues suspected to be parathyroid glands. Postoperative pathological reports were obtained as the golden standard to compare with the characteristic parameters obtained from bioelectrical impedance spectroscopy. The receiver operating characteristic curve analysis was used to assess the diagnostic value and the selection of the optimal threshold of these parameters from bioelectrical impedance spectroscopy.

**Results:**

A total of 512 patients were enrolled in the study and 1898 specimens were measured by the bioelectrical impedance spectroscopy analyzer. There were significant differences in the parameter of *f*_c_ among parathyroid glands, thyroid tissues, lymph nodes, and adipose tissues (252.2 ± 45.8 vs 144.7 ± 26.1, 491.7 ± 87.4, 602.3 ± 57.3; P<0.001, P<0.001, P<0.001). The area under the receiver operating characteristic curves was 0.993 (95%CI: 0.989-0.996) for *f*_c_. When the diagnostic criterion of *f*_c_ was set at 188.85 kHz~342.55 kHz, the sensitivity and specificity to identify parathyroid glands from lymph nodes and adipose tissues were both 100%. At this *f*_c,_ the sensitivity and specificity to identify parathyroid glands from thyroid tissues were 91.1% and 99.0%, respectively.

**Conclusion:**

In conclusion, bioelectrical impedance spectroscopy could assist to differentiate parathyroid glands from peripheral tissues during thyroid surgeries.

## Introduction

The incidence of thyroid neoplasm was increased in the past decades (1–6), and thyroid neoplasm ranks in ninth place for incidence and fifth for the most frequently diagnosed cancer in women worldwide (7). Thyroidectomy with central neck dissection has been widely adopted for the treatment of thyroid neoplasm (8–11). However, postoperative hypoparathyroidism is one of the main complications and the incidence ranges from 4.6% to 51.9% (12–14). Although the postoperative transient hypoparathyroidism may recover in a few months, it results in extended hospitalization and a poor experience for patients (15). In more severe cases, permanent hypoparathyroidism may occur and result in low quality of life (15–17).

Hypoparathyroidism is usually caused by parathyroid gland injury from mechanical or thermal trauma, devascularization, or removal (18–20). The surgeons need to do their best to protect parathyroid function by preserving the parathyroid gland in site with sufficient blood supply. Nevertheless, devascularization or accidental removal of parathyroid glands does happen despite of meticulous dissection in surgeries. In such cases, parathyroid gland autotransplantation is important to save the function of those parathyroid glands that could not be preserved in site (21–23). It is indisputable that functional recovery of the autotransplanted parathyroid gland depends on its survival rate (24, 25). Therefore, it is critical to keep the parathyroid glands fresh before transplantation. Because ischemia may denature parathyroid glands, autotransplantation should be performed as soon as possible. Currently, it takes at least 30 minutes for intraoperative frozen biopsy to identify accidentally removed parathyroid glands. Therefore, there is an unmet medical need to find other feasible and reliable methods to identify parathyroid glands in a shorter time during thyroid surgeries.

Electrical impedance is the measurement of resistance that a circuit poses to an alternating current when voltage is applied (26). The electrical properties of biological tissues in various frequency ranges are determined by the cellular components and structures, which generate characteristic impedance spectra (27). It was reported that electrical capacities were different between benign and malignant tumors in the breast (28). Electrical impedance of biological tissues is a complex data that combines resistance and capacitance. It depends on the frequency of the alternating current voltage applied to the tissues and reflects the tissue characteristics of conductivity and charge storage properties (27).

Bioelectrical Impedance Spectroscopy (BIS) had been applied to identify malignant lesions since 1984 (29). This technique had been widely used for the diagnosis of various carcinomas (30–34) and for characterizing various tissues (35, 36). As for thyroid neoplasm, there were several studies focused on the diagnosis of thyroid nodules (37–40). Antakia and colleagues used the BIS technique to identify parathyroid glands in neck central compartment surgeries of a rabbit model (26). In a study of 54 patients, Hillary et al. suggested the feasibility of using the technique to aid parathyroid identification and preservation during thyroid and/or parathyroid surgery (41). In the current study, we included 512 patients to investigate the effectiveness of BIS in the identification of human parathyroid glands from other soft tissues during thyroid surgeries.

## Patients and methods

### Patients

This study recruited patients who underwent thyroid surgeries for various thyroid diseases at the Department of Thyroid and Breast Surgery of Chengdu Third People’s Hospital from January 2018 to February 2020. The study protocol was approved by the Ethics Committee of Chengdu Third People’s Hospital for the use of human subjects in research. All patients were provided written informed consent.

### Methods

Different tissues exhibit different bioelectrical characteristics, thus bioimpedance can be adopted to characterize tissues. One popular method for tissue characterization is to fit spectroscopic data to the Cole-Cole model (42). The Cole equation models the behavior of permittivity and conductivity as a function of frequency:


$$Z(f)=R_{\infty }+\frac{R_{0}-R_{\infty }}{1+{\left(j\frac{f}{f_{c}}\right)}^{1-\alpha }}$$

**Figure 1:**
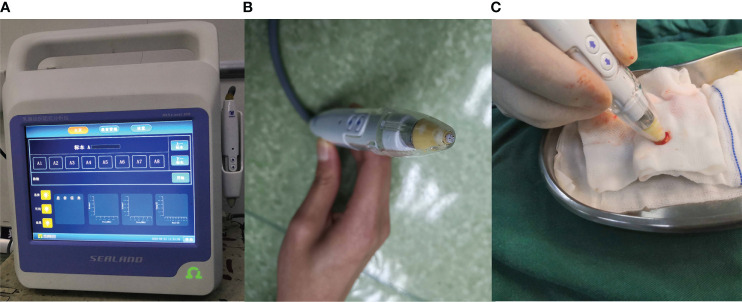
The Bioelectrical Impedance Spectroscopy analyzer. **(A)** analyzer. **(B)** probe. **(C)** the way of measurement.

Where *Z* (*f*) is the bioimpedance spectrum which is a vector of impedances; *f* is the spectrum frequency; R_∞_ is the impedance at infinite frequency; R_0_ is the impedance at direct current; *f*_c_ is the characteristic frequency of the tissue under analysis; α is the constant that characterizes the Cole distribution function. Data *Z* (*f*) are fitted by nonlinear regression curve fitting. The resulting model parameter vector for each tissue is m = [R_0_;R_∞_; *f*_c_; α]. The BIS analyzer (MScan1.0B, Sealand Technology, Chengdu, China; **Figure 1**) was used to measure the impedance of different specimens. The mechanism and measurement method of the BIS analyzer were also introduced by Du Z and Wu J (33, 34) and four parameters (R_0_, R_∞_, *f*_c,_ and α)were included in the analysis. The BIS analyzer was applied to the following tissues within two minutes after being resected during surgeries: thyroid glands, lymph nodes, adipose tissues, and tissues suspected to be parathyroid glands. Suspected parathyroid glands included true parathyroid glands that could not be preserved in site for surgical reasons and possible parathyroid glands confirmed to be lymph nodes and adipose tissues. Postoperative pathological reports were obtained by examining the paraffin sections and served as the golden standard to compare the BIS parameters.

### Statistical analysis

The statistical analysis was performed with SPSS version 23.0 software (SPSS Inc, Chicago, IL). The results of parameters of BIS were expressed as mean ± standard deviation (SD) Unpaired Student’s t-test was used to compare between groups. The receiver operating characteristic (ROC) curve analysis was used for the judgment of the diagnostic ability of the parameters and the selection of the optimal threshold value for diagnosis. The data of the tissues that were suspected to be parathyroid glands were used to verify the accuracy of the given diagnostic criteria. Statistical significance was set at P<0.05.

## Results

A total of 512 patients were enrolled in this study, among which 436 patients underwent thyroid surgeries for carcinoma, 43 patients for thyroid adenoma, and 33 patients for multinodular goiter. During surgeries, 1898 specimens were analyzed by the BIS analyzer, among which 362 specimens were suspected as parathyroid glands and were further examined by the intraoperative frozen section biopsy (**Table 1**). The results of postoperative pathology examinations and BIS parameters of different tissues were listed in **Table 2**. There were significant differences in the parameter *f*_c_ between parathyroid glands and thyroid tissues, lymph nodes, and adipose tissues. Similar differences were obtained in the parameters of R_0_/R_∞_ and α between parathyroid glands and thyroid tissues, lymph nodes, and adipose tissues (**Table 2**).

**Table 1:** The relationship between patients and measured tissues.

Measured tissues	Patients (n)
**1PG+1T+1LN+1AT**	214
**1PG+1T+1LN+2AT**	17
**1PG+1T+2LN+1AT**	5
**1T+1LN+1AT**	172
**1T+1LN+2AT**	34
**1T+2LN+1AT**	70

PG, parathyroid gland; T, thyroid; LN, lymph node; AT, adipose tissue.

**Table 2:** The parameters of BIS of different tissue.

Parameters	PG (n = 236)	T (n=512)	LN (n=587)	AT (n=563)	P_T_	P_LN_	P_AT_
***f*_c_ (kHz)**	252.2 ± 45.8	144.7 ± 26.1	491.7 ± 87.4	602.3 ± 57.3	<0.001	<0.001	<0.001
**R_0_/R_∞_**	4.44 ± 0.57	5.49 ± 0.86	7.01 ± 1.17	12.55 ± 1.44	<0.001	<0.001	<0.001
**α**	0.56 ± 0.06	0.67 ± 0.07	0.72 ± 0.07	0.84 ± 0.07	<0.001	<0.001	<0.001

BIS, bioelectrical impedance spectroscopy; PG, parathyroid gland; T, thyroid; LN, lymph node; AT, adipose tissue; P_T_: PG vs T; P_LN_ : PG vs LN; P_AT_ : PG vs AT.

**Figure 2:**
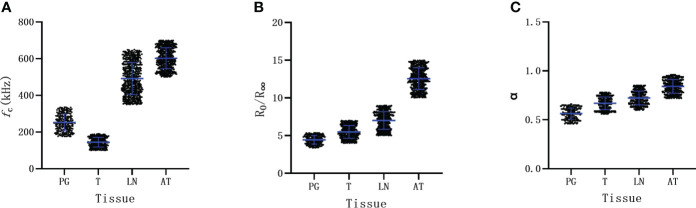
The distribution of Bioelectrical Impedance Spectroscopy parameters of different tissue. **(A)** parameter of *f*_c_. **(B)** parameter of R_0_/R_∞_. **(C)** parameter of α. PG parathyroid gland, T thyroid, LN lymph node, AT adipose tissue.

We further analyzed the distribution of BIS parameters among different tissues. There were significantly different distributions in *f*_c_ among these tissues (**Figure 2**). The *f*_c_ of the parathyroid gland slightly overlapped with that of the thyroid tissues, but there was no overlap between parathyroid glands and lymph nodes or adipose tissues (**Figure 2A**). The distribution of parameters of R_0_/R_∞_ and α of parathyroid glands partly overlapped with that of thyroid tissues as well as that of lymph nodes (**Figures 2B, C**).

**Figure 3:**
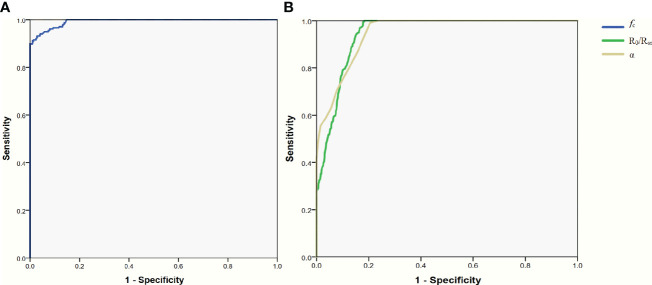
ROC curve for the parameters of Bioelectrical Impedance Spectroscopy. **(A)** parameter of *f*_c_. **(B)** parameter of R_0_/R_∞_ and α. ROC receiver operating characteristic.

The area under the ROC curves was 0.993 (95%CI: 0.989-0.996; **Figure 3A**), 0.944(95%CI: 0.934-0.954; **Figure 3B**) and 0.948(95%CI: 0.938-0.958; **Figure 3B**) for *f*_c_, R_0_/R_∞_, and α, respectively. According to these results, *f*_c_ was selected to further evaluate its diagnostic value. The mean value of *f*_c_ for parathyroid glands was larger than that for thyroid tissues (252.2 ± 45.8 vs 144.7 ± 26.1, P<0.001; **Table 2**), but was lower than that for lymph nodes and adipose tissues (252.2 ± 45.8 vs 491.7 ± 87.4, 602.3 ± 57.3; respectively, P<0.001, P<0.001; **Table 2**). The ranges of *f*_c_ did not overlap between parathyroid glands, lymph nodes, or adipose tissues (**Figure 2A**). The upper threshold of *f*_c_ was 342.55 kHz according to the ROC curve analysis (sensitivity 100%, specificity 100%). The lower threshold of *f*_c_ was 188.85 kHz (sensitivity 91.1%, specificity 99.0%; **Figure 3A**). The sensitivity and specificity were both 100% when the diagnostic criterion was used for identification of the tissues suspected to be parathyroid glands.

## Discussion

In this study, we confirmed that the bioelectrical impedances were different among the parathyroid glands, thyroid tissues, lymph nodes, and adipose tissues. The *f*_c_ had some overlap between the parathyroid glands and thyroid tissues, but it had no overlap between parathyroids and lymph nodes or adipose tissues. The R_0_/R_∞_ and α of the parathyroid glands were the lowest among those of the four types of tissue.

A possible explanation is that different tissues consist of different types of cells or different proportions of similar cells. The bioelectrical impedances of tissues are determined by the properties of cells, the patterns of how these cells were constructed, and the cellular constituents such as lipid content and nuclear size, etc. Based on a similar theory, a multiparametric imaging approach can help to differentiate the parathyroid gland from the thyroid (43). In this study, the BIS parameters of the parathyroid glands were proved to be significantly different from those of other tissues. The types of cells in tissues might be responsible for the differences in BIS parameters. As described by Antakia et al., a thyroid gland is composed mostly of follicles that store thyroglobulin, lined by a single layer of epithelial cells with calcitonin secreting parafollicular cells scattered between follicles (26). In contrast, a parathyroid gland is composed of densely packed chief and oxyphil cells. Although fine-needle aspiration cytology confirmed that there is significant overlap in the cytomorphologic features of cells derived from the parathyroid and thyroid gland, the parathyroid was significantly associated with vascular proliferation, bare nuclei, intracytoplasmic fat vacuolation, high cellularity, and the absence of colloid (44, 45). Our data suggested that the compositions of lymph nodes and adipose tissue were different from that of the parathyroid gland and thyroid, which manifested in their distinctive BIS parameters.

From the view of thyroid surgeons, the greatest challenge of identifying parathyroid gland is to distinguish the parathyroid glands from the adipose tissue with naked eyes because of their similar appearance. Occasionally, lymph nodes might also be mistakenly identified as parathyroid glands. The use of marking techniques, such as carbon nanoparticle, methylene blue, and near-infrared imaging, provides some degree of help to distinguish these tissues (46–48). However, parathyroid glands are always accompanied or wrapped by adipose tissues, which causes the greatest challenge of identification.

In recent decades, more marking instruments have become available. Pasta and coworkers reported that Technetium (Tc99^m^)-sestaMIBI was a helpful radiotracer for intraoperative localization of the parathyroid glands (49). Methylene blue was tested as a tracer for parathyroid identification, and the carbon nanoparticle was also used as a marking method (46). Carbon nanoparticle is a lymphatic tracer that stains and distinguishes the thyroid and lymphatic system, while the parathyroid and adipose tissues can be differentiated by being untinged. Near-infrared auto-fluorescence spectroscopy was also reported to be able to identify the parathyroid glands during thyroid surgeries (48). However, such methods still require the experience of surgeons to distinguish parathyroid glands from adipose tissues.

During surgeries, surgeons often preserve the suspected parathyroid glands in site, but the nature of these suspected parathyroid glands may not be verified by pathology examination. In this study, we used a BIS analyzer to analyze those suspected parathyroid glands that could not be preserved in site or found in the intraoperative resected specimens. The natures of these suspected parathyroid glands were verified by intraoperative frozen pathological examination. When the parathyroid glands were confirmed by intraoperative frozen pathological diagnoses, they were transplanted into the sternocleidomastoid muscle. One of the most difficult issues is that intraoperative frozen pathological diagnoses always takes more than 30 minutes to get the reports. In contrast, BIS analyses only took a few minutes, and our results showed that the parameter *f*_c_ had perfect accuracy in distinguishing the parathyroid gland from lymph node and adipose tissue.

Our study opens new avenues for further research on this line. For example, the bioelectrical impedances could be sensitive to environment temperature and humidity. Whether these environmental changes may affect the cellular constituents such as lipid content and nuclear size needs further investigation. Some multicenter trials with larger samples are necessary to establish more reliable and accurate criteria.

## Conclusions

In summary, this study indicates that the bioelectrical impedances vary from tissue to tissue. BIS is a potentially powerful tool to assist in identifying the parathyroid gland during thyroid surgeries. The results of this study would help reduce the incidence of postoperative hypoparathyroidism and thus help the recovery and quality of life of patients.

## Data availability statement

The original contributions presented in the study are included in the article/supplementary material. Further inquiries can be directed to the corresponding author.

## Ethics statement

The studies involving human participants were reviewed and approved by Ethics Committee of Chengdu Third People’s Hospital. The patients/participants provided their written informed consent to participate in this study.

## Author contributions

BW and JiW performed the research. All the authors contributed to the design of the work and the acquisition and interpretation of data. BW performed the statistical analysis and wrote the first draft. ZL revised the first draft. All authors contributed to the further drafts. JiW is the guarantor.

## Funding

BW was supported by a nonprofit fund from China Health Promotion Foundation. JiW was supported by a grant from Scientific Research Fund of the Department of Science and Technology of Chengdu City (2015-HM01-00376-SF) and Science and Technology Program of Science & Technology Department of Sichuan Province (2015JY0190). The funding bodies had no role in the conception of the study, in the collection, analysis, and interpretation of data, in writing the manuscript and in the approval of the publication.

## Acknowledgments

The authors thank the patients for their participation and the Sealand Technology (Chengdu) Limited for providing the BIS instrument.

## Conflict of interest

The authors declare that the research was conducted in the absence of any commercial or financial relationships that could be construed as a potential conflict of interest.

## Publisher’s note

All claims expressed in this article are solely those of the authors and do not necessarily represent those of their affiliated organizations, or those of the publisher, the editors and the reviewers. Any product that may be evaluated in this article, or claim that may be made by its manufacturer, is not guaranteed or endorsed by the publisher.

## References

[1] Leenhardt L Bernier MO Boin-Pineau MH Conte Devolx B Marechaud R Niccoli-Sire P. Advances in diagnostic practices affect thyroid cancer incidence in France. Eur J Endocrinol (2004) 150(2):133–9. doi: 10.1530/eje.0.1500133 14763910

[2] Jung KW Won YJ Kong HJ Oh CM Seo HG Lee JS. Cancer statistics in Korea: incidence, mortality, survival and prevalence in 2010. Cancer Res Treat (2013) 45(1):1–14. doi: 10.4143/crt.2013.45.1.1 23613665 PMC3629358

[3] Du L Li R Ge M Wang Y Li H Chen W. Incidence and mortality of thyroid cancer in China, 2008-2012. Chin J Cancer Res (2019) 31(1):144–51. doi: 10.21147/j.issn.1000-9604.2019.01.09 PMC6433579 30996572

[4] Dal Maso L Lise M Zambon P Falcini F Crocetti E Serraino D. Incidence of thyroid cancer in Italy, 1991-2005: Time trends and age-period-cohort effects. Ann Oncol (2011) 22(4):957–63. doi: 10.1093/annonc/mdq467 20952599

[5] Qian ZJ Jin MC Meister KD Megwalu UC. Pediatric thyroid cancer incidence and mortality trends in the united states, 1973-2013. JAMA Otolaryngol. Head Neck Surg (2019) 145(7):617–23. doi: 10.1001/jamaoto.2019.0898 PMC6547136 31120475

[6] Lim H Devesa SS Sosa JA Check D Kitahara CM. Trends in thyroid cancer incidence and mortality in the united states, 1974-2013. JAMA (2017) 317(13):1338–48. doi: 10.1001/jama.2017.2719 PMC8216772 28362912

[7] Sung H Ferlay J Siegel RL Laversanne M Soerjomataram I Jemal A. Global cancer statistics 2020: GLOBOCAN estimates of incidence and mortality worldwide for 36 cancers in 185 countries. CA Cancer J Clin (2021) 71(3):209–49. doi: 10.3322/caac.21660 33538338

[8] Haugen BR Alexander EK Bible KC Doherty GM Mandel SJ Nikiforov YE. 2015 American thyroid association management guidelines for adult patients with thyroid nodules and differentiated thyroid cancer: The American thyroid association guidelines task force on thyroid nodules and differentiated thyroid cancer. Thyroid (2016) 26(1):1–133. doi: 10.1089/thy.2015.0020 26462967 PMC4739132

[9] American Thyroid Association Guidelines Task Force Kloos RT Eng C Evans DB Francis GL Gagel RF. Medullary thyroid cancer: Management guidelines of the American thyroid association. Thyroid (2009) 19(6):565–612. doi: 10.1089/thy.2008.0403 19469690

[10] Wells SA Jr. Asa SL Dralle H Elisei R Evans DB Gagel RF. Revised American thyroid association guidelines for the management of medullary thyroid carcinoma. Thyroid (2015) 25(6):567–610. doi: 10.1089/thy.2014.0335 25810047 PMC4490627

[11] American Thyroid Association Guidelines Taskforce on Thyroid Nodules Differentiated Thyroid Cancer Cooper DS Doherty GM Haugen BR Kloos RT Lee SL. Revised American thyroid association management guidelines for patients with thyroid nodules and differentiated thyroid cancer. Thyroid (2009) 19(11):1167–214. doi: 10.1089/thy.2009.0110 19860577

[12] Giordano D Valcavi R Thompson GB Pedroni C Renna L Gradoni P. Complications of central neck dissection in patients with papillary thyroid carcinoma: Results of a study on 1087 patients and review of the literature. Thyroid (2012) 22(9):911–7. doi: 10.1089/thy.2012.0011 22827494

[13] Lee YS Kim SW Kim SW Kim SK Kang HS Lee ES. Extent of routine central lymph node dissection with small papillary thyroid carcinoma. World J Surg (2007) 31(10):1954–9. doi: 10.1007/s00268-007-9171-7 17687598

[14] Pereira JA Jimeno J Miquel J Iglesias M Munne A Sancho JJ. Nodal yield, morbidity, and recurrence after central neck dissection for papillary thyroid carcinoma. Surgery (2005) 138(6):1095–100. doi: 10.1016/j.surg.2005.09.013 16360396

[15] Bhattacharyya N Fried MP. Assessment of the morbidity and complications of total thyroidectomy. Arch Otolaryngol. Head Neck Surg (2002) 128(4):389–92. doi: 10.1001/archotol.128.4.389 11926912

[16] Bohrer T Pasteur I Lyutkevych O Fleischmann P Tronko M. [Permanent hypoparathyroidism due to thyroid cancer surgical procedures in patients exposed to radiation in the Chernobyl, Ukraine, nuclear reactor accident]. Dtsch Med Wochenschr (2005) 130(44):2501–6. doi: 10.1055/s-2005-918594 16252209

[17] Polistena A Monacelli M Lucchini R Triola R Conti C Avenia S. Surgical morbidity of cervical lymphadenectomy for thyroid cancer: A retrospective cohort study over 25 years. Int J Surg (2015) 21:128–34. doi: 10.1016/j.ijsu.2015.07.698 26253851

[18] Su A Gong Y Wu W Gong R Li Z Zhu J. Does the number of parathyroid glands autotransplanted affect the incidence of hypoparathyroidism and recovery of parathyroid function? Surgery (2018) 164(1):124–9. doi: 10.1016/j.surg.2017.12.025 29398031

[19] Su A Gong Y Wu W Gong R Li Z Zhu J. Effect of autotransplantation of a parathyroid gland on hypoparathyroidism after total thyroidectomy. Endocr Connect (2018) 7(2):286–94. doi: 10.1530/EC-17-0313 PMC8111321 29301864

[20] Fahad Al-Dhahri S Al-Ghonaim YA Sulieman Terkawi A. Accuracy of postthyroidectomy parathyroid hormone and corrected calcium levels as early predictors of clinical hypocalcemia. J Otolaryngol. Head Neck Surg (2010) 39(4):342–8. 20642997

[21] Lahey FH. The transplantation of parathyroids in partial thyroidectomy. Surgery Gynecol Obstet (1926) 62:508–9.

[22] Palazzo FF Sywak MS Sidhu SB Barraclough BH Delbridge LW. Parathyroid autotransplantation during total thyroidectomy–does the number of glands transplanted affect outcome? World J Surg (2005) 29(5):629–31. doi: 10.1007/s00268-005-7729-9 15827848

[23] Sokouti M Montazeri V Golzari S. The incidence of transient and permanent hypocalcemia after total thyroidectomy for thyroid cancer. Int J Endocrinol Metab (2010) 8(1):7–12.

[24] Senapati A Young AE. Parathyroid autotransplantation. Br J Surg (1990) 77(10):1171–4. doi: 10.1002/bjs.1800771027 2224467

[25] Kihara M Miyauchi A Kontani K Yamauchi A Yokomise H. Recovery of parathyroid function after total thyroidectomy: Long-term follow-up study. ANZ J Surg (2005) 75(7):532–6. doi: 10.1111/j.1445-2197.2005.03435.x 15972040

[26] Antakia R Brown BH Highfield PE Stephenson TJ Brown NJ Balasubramanian SP. Electrical impedance spectroscopy to aid parathyroid identification and preservation in central compartment neck surgery: A proof of concept in a rabbit model. Surg Innov (2016) 23(2):176–82. doi: 10.1177/1553350615607639 26423912

[27] Dean DA Ramanathan T Machado D Sundararajan R. Electrical impedance spectroscopy study of biological tissues. J Electrostat (2008) 66(3-4):165–77. doi: 10.1016/j.elstat.2007.11.005 PMC2597841 19255614

[28] Crile GW. Measurements of electrical capacity of benign and malignant tumors-clinical significance. J Cancer Res (1925) 9(3):388–90. doi: 10.1158/jcr.1925.388

[29] Chaudhary SS Mishra RK Swarup A Thomas JM. Dielectric properties of normal & malignant human breast tissues at radiowave & microwave frequencies. Indian J Biochem Biophys (1984) 21(1):76–9. 6490065

[30] Smallwood RH Keshtkar A Wilkinson BA Lee JA Hamdy FC. Electrical impedance spectroscopy (EIS) in the urinary bladder: The effect of inflammation and edema on identification of malignancy. IEEE Trans Med Imaging (2002) 21(6):708–10. doi: 10.1109/TMI.2002.800608 12166869

[31] Aberg P Birgersson U Elsner P Mohr P Ollmar S. Electrical impedance spectroscopy and the diagnostic accuracy for malignant melanoma. Exp Dermatol (2011) 20(8):648–52. doi: 10.1111/j.1600-0625.2011.01285.x 21539620

[32] Halter RJ Schned A Heaney J Hartov A Schutz S Paulsen KD. Electrical impedance spectroscopy of benign and malignant prostatic tissues. J Urol (2008) 179(4):1580–6. doi: 10.1016/j.juro.2007.11.043 18295258

[33] Du Z Wan H Chen Y Pu Y Wang X. Bioimpedance spectroscopy can precisely discriminate human breast carcinoma from benign tumors. Med (Baltimore) (2017) 96(4):e5970. doi: 10.1097/MD.0000000000005970 PMC5287972 28121948

[34] Wu J Wang P Tang Y Liu H Wang H Zhang W. Technical note: A new method to rapidly identify benign and malignant breast lumps through bioelectrical impedance spectroscopy. Med Phys (2019) 46(5):2522–5. doi: 10.1002/mp.13474 30859583

[35] Rigaud B Hamzaoui L Frikha MR Chauveau N Morucci JP. *In vitro* tissue characterization and modelling using electrical impedance measurements in the 100 Hz-10 MHz frequency range. Physiol Meas (1995) 16(3 Suppl A):A15–28. doi: 10.1088/0967-3334/16/3a/002 8528113

[36] Tao D Adler A. *In Vivo*Blood characterization from bioimpedance spectroscopy of blood pooling. IEEE Trans Instrum Meas (2009) 58(11):3831–8. doi: 10.1109/tim.2009.2020836

[37] Cheng Y Fu M. Dielectric properties for differentiating normal and malignant thyroid tissues. Med Sci Monit (2018) 24:1276–81. doi: 10.12659/msm.908204 PMC5844190 29499032

[38] Stojadinovic A Fields SI Shriver CD Lenington S Ginor R Peoples GE. Electrical impedance scanning of thyroid nodules before thyroid surgery: A prospective study. Ann Surg Oncol (2005) 12(2):152–60. doi: 10.1245/ASO.2005.03.062 15827796

[39] Nissan A Peoples GE Abu-Wasel B Adair CF Prus D Howard RS. Prospective trial evaluating electrical impedance scanning of thyroid nodules before thyroidectomy: Final results. Ann Surg (2008) 247(5):843–53. doi: 10.1097/SLA.0b013e318165c757 18438123

[40] Zheng B Tublin ME Klym AH Gur D. Classification of thyroid nodules using a resonance-frequency-based electrical impedance spectroscopy: A preliminary assessment. Thyroid (2013) 23(7):854–62. doi: 10.1089/thy.2012.0413 PMC3704105 23259723

[41] Hillary SL Brown BH Brown NJ Balasubramanian SP. Use of electrical impedance spectroscopy for intraoperative tissue differentiation during thyroid and parathyroid surgery. World J Surg (2020) 44(2):479–85. doi: 10.1007/s00268-019-05169-7 31511942

[42] Cole KS Cole RH. Dispersion and absorption in dielectrics I. Alternating current characteristics. J Chem Phys (1941) 9(4):341–51. doi: 10.1063/1.1750906

[43] Isidori AM Cantisani V Giannetta E Diacinti D David E Forte V. Multiparametric ultrasonography and ultrasound elastography in the differentiation of parathyroid lesions from ectopic thyroid lesions or lymphadenopathies. Endocrine (2017) 57(2):335–43. doi: 10.1007/s12020-016-1116-1 27709473

[44] Dimashkieh H Krishnamurthy S. Ultrasound guided fine needle aspiration biopsy of parathyroid gland and lesions. Cytojournal (2006) 3:6. doi: 10.1186/1742-6413-3-6 16569241 PMC1435923

[45] Kumari N Mishra D Pradhan R Agarwal A Krishnani N. Utility of fine-needle aspiration cytology in the identification of parathyroid lesions. J Cytol (2016) 33(1):17–21. doi: 10.4103/0970-9371.175490 27011436 PMC4782397

[46] Li Y Jian WH Guo ZM Li QL Lin SJ Huang HY. A meta-analysis of carbon nanoparticles for identifying lymph nodes and protecting parathyroid glands during surgery. Otolaryngol. Head Neck Surg (2015) 152(6):1007–16. doi: 10.1177/0194599815580765 25897006

[47] Su AP Wei T Gong YP Gong RX Li ZH Zhu JQ. Carbon nanoparticles improve lymph node dissection and parathyroid gland protection during thyroidectomy: A systematic review and meta-analysis. Int J Clin Exp Med (2018) 11(2):463–73.

[48] Liu J Wang X Wang R Xu C Zhao R Li H. Near-infrared auto-fluorescence spectroscopy combining with fisher's linear discriminant analysis improves intraoperative real-time identification of normal parathyroid in thyroidectomy. BMC Surg (2020) 20(1):4. doi: 10.1186/s12893-019-0670-x 31907042 PMC6945439

[49] Pasta V Monteleone F Del Vecchio L Iacobelli S Urciuoli P D'Orazi V. Original technique for preoperative preparation of patients and intraoperative localization of parathyroid adenomas. G Chir (2015) 36(3):97–100. doi: 10.11138/gchir/2015.36.3.097 26188752 PMC4511047

